# Coprs inactivation leads to a derepression of *LINE1* transposons in spermatocytes

**DOI:** 10.1002/2211-5463.12562

**Published:** 2018-12-19

**Authors:** Conception Paul, Hélène Delpech, Delphine Haouzi, Samir Hamamah, Claude Sardet, Eric Fabbrizio

**Affiliations:** ^1^ Institut de Génétique Moléculaire de Montpellier UMR5535 CNRS Montpellier University France; ^2^ Institut de Recherche en Cancérologie de Montpellier U1194 Inserm, ICM, CNRS Montpellier University Montpellier Cedex 5 France; ^3^ ART‐PGD Department Institute of Regenerative Medicine and Biotherapy CHU Montpellier Inserm U1203 UFR of Medicine Saint‐Eloi Hospital Montpellier University France

**Keywords:** coprs, Miwi, piRNA, spermatocyte, teratozoospermia, testis

## Abstract

Repression of retrotransposons is essential for genome integrity during germ cell development and is tightly controlled through epigenetic mechanisms. In primordial germ cells, protein arginine *N*‐methyltransferase (Prmt5) is involved in retrotransposon repression by methylating Piwi proteins, which is part of the piRNA pathway. Here, we show that in mice, genetic inactivation of *coprs* (which is highly expressed in testis and encodes a histone‐binding protein required for the targeting of Prmt5 activity) affects the maturation of spermatogonia to spermatids. Mass spectrometry analysis revealed the presence of Miwi in testis protein lysates immunoprecipitated with an anti‐Coprs antibody. The observed deregulation of Miwi and pachytene pre‐piRNAs levels and the derepression of *LINE1* repetitive sequences observed in *coprs*‐/‐ mice suggest that Coprs is implicated in genome surveillance mechanisms.

**Abbreviations**
Coprs: coordinator of Prmt5 and differentiation stimulator
IAP: intracisternal A‐particle
LINE1: long interspersed nuclear element 1
piRNA: Piwi‐interacting RNA
Prmt5: protein arginine *N*‐methyltransferase
SINE: short interspersed nuclear element

Spermatogenesis is a multistep process that takes place in the seminiferous tubules whereby mature spermatozoa are continuously produced during adulthood reproduction lifetime. Specifically, primordial germ cells (PGCs) generate spermatogonia that either self‐renew to maintain a pool of stem cells or undergo differentiation. After two meiotic divisions, secondary spermatocytes are produced. Then, during spermiogenesis, they differentiate into elongating spermatids and finally spermatozoa 1, 2, 3, 4, 5. Importantly, these changes are associated with chromatin compaction, transcription arrest, and specific epigenetic modifications that contribute to the production of mature sperm. Concomitantly, to maintain genome integrity, PGCs need to repress repetitive DNA elements because their spread can lead to inherited diseases. These repetitive sequences [for instance, long interspersed nuclear elements 1 (LINEs), short interspersed nuclear element (SINEs), and intracisternal A‐particle (IAP) sequences] derive mostly from transposable elements and represent about 40% of the human genome 3, 6, 7, 8. Thus, their repression is challenging and is tightly controlled in germ cells by the Piwi‐interacting RNA (piRNA) pathway and through epigenetic mechanisms 9, 10. In the mouse, this pathway involves the three Piwi proteins Miwi, Mili, and Miwi2 (PIWIL1, PIWIL2, and PIWIL4 in humans, respectively) that show different developmental expression patterns in testis 11, 12. *Mili* is expressed in fetal germ cells up to the round spermatid stage, while *Miwi2* expression is restricted to fetal and perinatal germ cells. In adult testes, *Miwi* is expressed in pachytene spermatocytes up to round spermatid testes 13. Mili and Miwi bind to both piRNAs in pachytene spermatocytes and postmeiotic spermatids 14. Deletion of the *Miwi* gene arrests cells at the round spermatid stage, and *Mili* deficiency leads to male sterility 14. Chromatin‐modifying enzymes also participate in the regulation of these repetitive elements. For instance, protein arginine *N*‐methyltransferase 5 (Prmt5) is implicated in the piRNA pathway in PGCs via a Prmt5‐dependent methylation of Piwi proteins. In somatic cells, Prmt5 modulates cell proliferation and differentiation, for instance, during myogenesis and adipogenesis, by interacting with various proteins, including Coprs (previously termed Copr5), a histone‐binding protein 15, 16, 17, 18, 19, 20, 21, 22, 23, 24. In addition, Prmt5‐methylated arginine residues can be bound by proteins harboring a Tudor domain. In PGCs, many of these proteins (e.g., Piwi proteins) are essential for male fertility 25. It was suggested that Prmt5 maintains DNA integrity during global epigenetic reprogramming and that its subcellular localization affects the piRNA pathway, at least in part, and promotes transposon silencing 26. In addition*,* conditional loss of *Prmt5* in early mouse PGCs causes complete male and female sterility that was attributed to global impairment of DNA demethylation in the genome 26.

Here, we show that in *coprs*‐/‐ mice, maturation of spermatogonia to spermatids is affected, although animals are fertile. Moreover, the mRNA levels of *Ccna1* (cyclin A1*)*,* Prm1* (sperm protamine P1), *Miwi*, pachytene piRNA precursors, and *LINE1* are deregulated in *coprs*‐/‐ testes, suggesting that Coprs contributes to genome surveillance mechanisms via the Piwi‐piRNA pathway.

## Materials and methods

### Flow cytometry

Extraction of cells in testis was performed essentially as described in Mays‐Hoopes *et al*. 27. Testes from wild‐type (WT *n* = 3) and knockout (KO *n* = 4) mice were dissected and decapsulated to release the tubules. Seminiferous tubules were incubated with 0.25 mg·mL^−1^ collagenase type IV (Sigma) at 37 °C under rapid agitation for 5 min and washed to release Leydig cells and interstitial cells. Dispersed tubules were allowed to settle and washed twice to remove peritubular cells. Washed tubules were then incubated with 0.5% trypsin/EDTA (Gibco) and 1 μg·mL^−1^ DNase RQ1 (Promega) at 37 °C for 5 min. Trypsin digestion was stopped by adding DMEM with 10% FBS. Suspensions were washed and disaggregated into single‐cell suspensions by trituration before filtration through a 50‐μm cell strainer. For analysis, cells were fixed in 0.4 m citrate buffer (pH 4.5) overnight and resuspended in 1 mL of cold propidium iodide (PI) staining solution (10 mm Tris/HCl (pH 8.0), 1 mm NaCl, 0.1% Nonidet P‐40, 50 μg·mL^−1^ PI, 10 μg·mL^−1^ RNase A), vortexed, and incubated on ice for 10 min to lyse the plasma membrane and stain nuclear DNA. DNA content was assessed on a FACSCalibur II (Becton Dickinson) equipped with the cellquest software.

### Western blotting

Anti‐Prmt5 (Millipore) and anti‐Miwi (Abcam) antibodies were used according to the manufacturer's instructions. The anti‐Coprs antibody (AGRO‐BIO, Clermont‐Ferrand, France) was against the last 20 amino acids of Coprs C terminus 21.

### Mass spectrometry analysis

In‐gel digestion of bands excised from Colloidal Blue‐stained gel was done before LC/MS/MS analysis that was performed at the Taplin Mass Spectrometry Facility (Harvard Medical School, Boston).

### Immunohistochemistry (IHC)

Tissues were fixed in Bouin's fixative and embedded in paraffin. Then, 4‐μm‐thick sections were cut and processed for IHC staining. IHC was performed using the same anti‐Coprs and anti‐Miwi (Abcam) antibodies employed for western blotting, followed by a biotinylated secondary antibody coupled to the streptavidin–peroxidase complex (ABC Vectastain Kit; Vector Laboratories). Revelation was performed with the peroxidase substrate DAB (3,3′‐diaminobenzidine) from Vector Laboratories.

### RNA isolation, cDNA synthesis, and RT‐qPCR amplification from mice

Total semen RNA was isolated with the TRIzol Reagent (Life Technologies) according to the manufacturer's instructions. Briefly, 800 μL of TRIzol and 200 μL of chloroform were added to 200 μL of sperm. The mixture was mixed for 15 s and left at room temperature for 5 min. After centrifugation at 12 000 ***g*** at 4 °C for 15 min, supernatants were transferred to fresh tubes containing 1 volume equivalent of 70% ethanol. Then, total RNA was purified using the miRNeasy Serum/Plasma Kit (Qiagen) according to the manufacturer's recommendations. RNA was quantified with a NanoDrop ND‐1000 spectrophotometer (NanoDrop Technologies Inc., DE, USA). RNA isolation from mouse testes and RT‐qPCR were performed as described 18. Briefly, testes were lysed in TRIzol reagent (Invitrogen), and total RNA was isolated according to the manufacturer's recommendations. cDNA was synthesized from 1 μg of total RNA using random hexamers and SuperScript III Reverse Transcriptase (Invitrogen). Real‐time qPCR was performed on a LightCycler 480 SW 1.5 apparatus (Roche) with Platinum Taq DNA Polymerase (Invitrogen) in the presence and the SYBR Green Mix.

### Oligonucleotide sequences

*Oct4 f* CAATGAGAACCTTCAGGAGATATGC, r TCAATGCTAGTTCGCTTTCTCTTC;

*Amh f* TAGTCCTACATCTGGCTGAAGTGATATG, r CCAGGTGGAGGCTCTTGGA;

*Ccna1 f* CACTTCCTGCTGGATTTCAAC, r CGATGAATCTCCTCTGCATAC;

*Prm1 f* GCCGCTCATACACCATAAGG, r CAAGATGTGGCGAGATGC;

*Prm2 f* GCAGAAGATCCCGAAGGAG, r CTCCAGGCAGAATGGACAG;

*H1t2* f AGGGAAGAGAAGGGACAGGA, r CTTGGAGCCCATATGGAAAA;

*Hils*1 f GTCCCAAGCCAGAGTGAGAG, r CTTGAAGCGCCAGGTGTTAT;

*Miwi* f ATGATCGTGGGCAT, r AGGCCACTGCTGTCATA;

*pre‐piR1* f GTTAGCGAAGGACATTATTCTAACC, r TGACATGAACACAGGTGCTCAGAT;

*pre‐piR2* f CTATGCTTATGATGGCATTGGAGAG, r TTCCAGTTCAACAGGGACACGGGAC;

*pre‐piR3* f GTTCTCACTTTATCAGCTCTCAAG, r TGAGAGTGGCATCTAAATGTTTAG;

*Line1* f GGAGGGACATTTCATTCTCATCA, r GCTGCTCTTGTATTTGGAGCATAGA;

*SineB1* f GTGGCGCACGCCTTTAATC, r GACAGGGTTTCTCTGTGTAG;

*IAP* f ATTGTTCCCTCACTGGCAAA, r ATTGTTCCCTCACTGGCAAA;

*Actin* f AGAAGAGCTATGAGCTGCCT, r TCATCGTACTCCTGCTTGCT;

*hCOPRS f TCCGCCTCCACTGATACCC,* r GGCCCGCCCACCTACAA*;*

*hHPRT* f TGACACTGGCAAAACAATGCA, r GGTCCTTTTCACCAGCAAGCT.

### Mice and animal care

Animal experiments were approved by the Ethics Committee of the Languedoc‐Roussillon Region (France).

### Patient's characterization

Nine patients were recruited from our IVF/ICSI program at the ART‐PGD Department of Montpellier University Hospital, France, after signature of the written informed consent. Each patient was classified according to the fraction (%) of typical spermatozoon forms (TF) evaluated using one of three classical methods 28, 29, 30. The study methodologies were approved by the local ethics committee and conformed to the standards set by the Declaration of Helsinki.

## Results

### Coprs is localized in spermatogonia and its genetic inactivation leads to male germ cell accumulation at the pachytene stage

**Figure 1:**
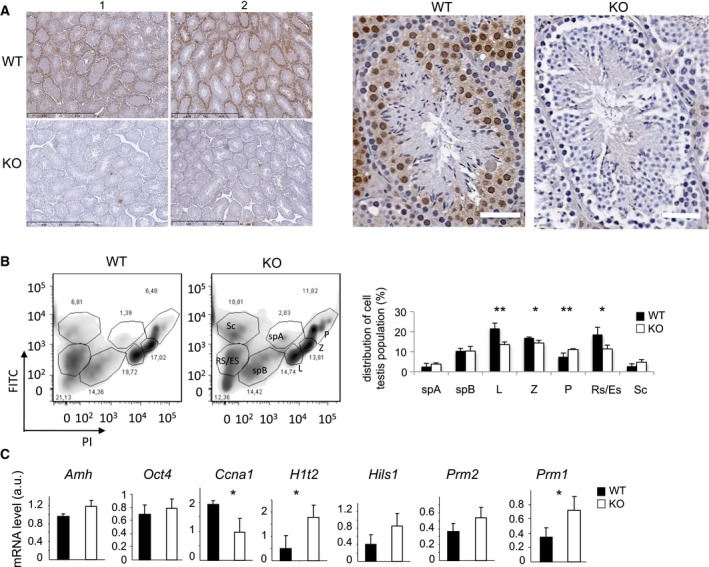
In mice, Coprs is localized in spermatogonia and its absence delays spermatid maturation. (A) Left panel: IHC staining of WT (*n* = 2) and KO (*n* = 2) testis sections with an anti‐Coprs antibody is presented. Bar = 1 mm. Right panel: images at higher resolution. Bar = 50 μm. (B) Flow cytometry analysis (left panels) and histograms (right panel) showing the distribution of the different mouse testis cell types. Sc: Sertoli cells; Rs/Re: round/elongated spermatids; SpB: type B spermatogonia; SpA: type A spermatogonia; L, Z, and P: leptotene, zygotene, and pachytene spermatocytes. Results are expressed as the percentage of each cell type relative to all counted cells and are the mean ± SEM (*n* = 3 WT;* n* = 4 KO). *P < 0.1 and **P < 0.05 (Student's *t*‐test). (C) RT‐qPCR analysis of the expression of the indicated genes in WT and KO testes. Expression was normalized to *actin* expression, and values are expressed in arbitrary units (a.u.) and are the mean ± SEM of RNA samples independently prepared (*n* = 3 WT;* n* = 4 KO). *P < 0.05 (Student's *t*‐test).

Our previous studies 21 and the available Gene Expression Omnibus (GEO) data on *Coprs* expression profile in various human tissues confirmed that it was strongly expressed in testis (Fig. S1). To investigate Coprs role in testis, we used *coprs* KO mouse 22. Morphological analysis of 8‐ and 36‐week‐old *coprs* KO males did not show testicular atrophy or reduced body weight (data not shown). IHC analysis of paraffin‐embedded testis sections prepared from *coprs* KO animals and control littermates showed that Coprs was expressed only in germ cells and was mostly localized in the nucleus, although the anti‐Coprs antibody stained also faintly the cytoplasmic compartment (Fig. 1A; note the absence of expression in *coprs* KO testis). A similar nuclear localization was previously observed in different culture cell types 21. As we and others previously reported that Coprs is implicated in cell differentiation 16, 17, 18, 22, we analyzed germ cell maturation and the distribution of the different cell types in WT and KO testes by flow cytometry. The proportion of germ cells that progressed through meiosis in the leptotene and zygotene stages was reduced in KO mice, and cells accumulated at the pachytene stage (Fig. 1B). Moreover, the proportion of round and elongated spermatids was lower in *coprs* KO than WT testes (Fig. 1B). These data suggest that Coprs absence perturbs spermatogenesis between the end of meiosis and spermiogenesis.

To further document at what stage *coprs* inactivation impaired the maturation of spermatogonia, we measured by RT‐qPCR the expression level of various differentiation markers in testes of 8‐week‐old mice. The mRNA levels of *Oct4* and *Amh*, two markers of undifferentiated spermatogonia and Sertoli cells, respectively, were not significantly different between WT and KO samples (Fig. 1C). This suggested that the initial pool of undifferentiated spermatogonia was comparable in WT and KO animals. In agreement with the flow cytometry data, the mRNA levels of the premeiotic marker *Ccna1* were downregulated, whereas the level of the histone variant *H1t2*, which is usually expressed in pachytene spermatocytes and persists in early spermatids, was upregulated in *coprs* KO testes (Fig. 1C). Similarly, *Prm1* mRNA level was significantly increased in *coprs* KO testis cells (Fig. 1C). In contrast, spermatid‐specific linker histone *Hils1* and *Prm2* levels did not vary significantly between WT and KO in elongated spermatids (Fig. 1C). Consequently, the *Prm1*/*Prm2* ratio, an indicator of chromatin compaction 31, was significantly higher in *coprs* KO testes than in controls (1.34 in *coprs* KO mice vs 0.92 in WT animals), suggesting that Coprs deficiency results in perturbations of chromatin compaction.

These data indicated that *coprs* is implicated in spermatocyte maturation.

### Coprs gene ablation leads to deregulation of the piRNA pathway and derepression of LINE1 repetitive elements

**Figure 2:**
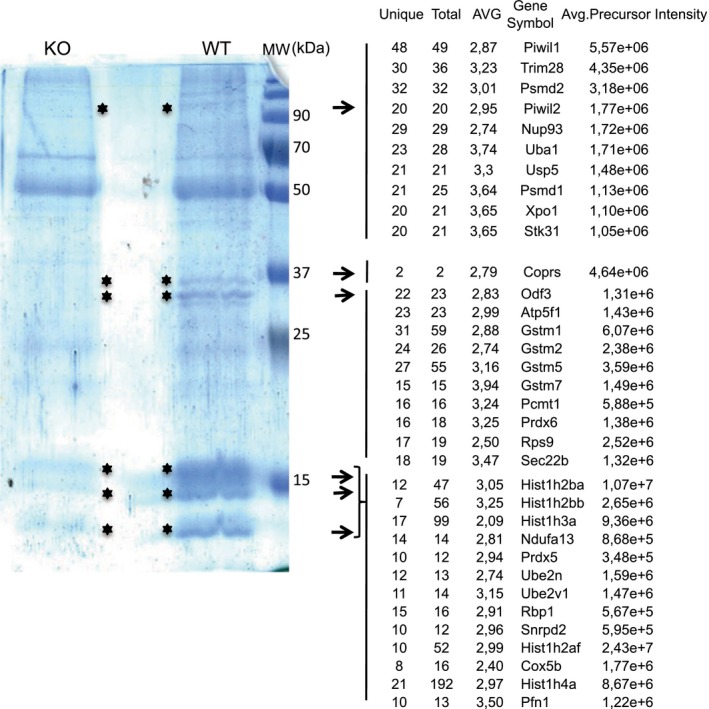
Identification of Miwi as a major protein immunoprecipitated with Coprs. Protein extracts from WT and *coprs* KO testes were immunoprecipitated with an anti‐Coprs antibody. Bands (*) present only in the WT lane were excised from the Colloidal Blue‐stained gel (left) and analyzed by mass spectrometry (right).

**Figure 3:**
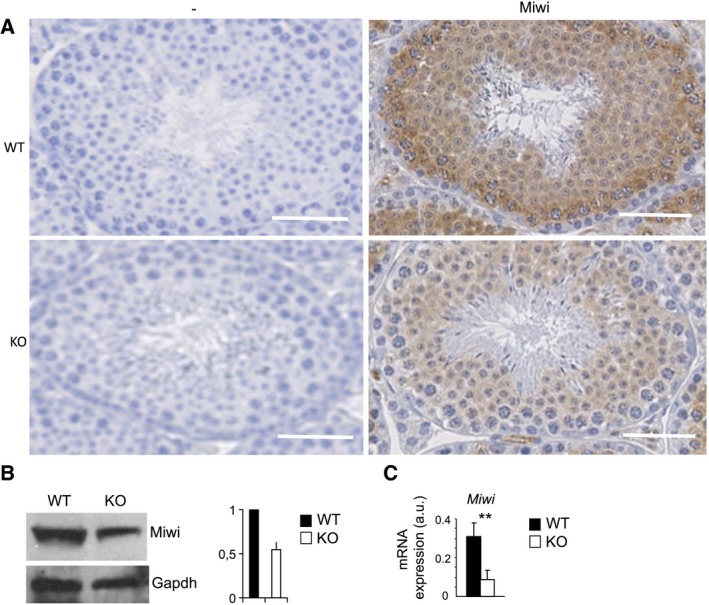
*coprs* KO affects Miwi expression level. (A) IHC analysis of WT and KO mouse testis sections with or without (negative control, ‐) an anti‐Miwi antibody; bar = 50 μm. (B) Left panel: western blot detection of Miwi expression in whole‐cell extracts from WT and KO testes with anti‐Miwi and anti‐Gapdh antibodies; right panel: quantification of Miwi expression with ImageJ after normalization to Gapdh expression. Values are expressed in arbitrary units (a.u.) and are the mean ± SEM of two independent experiments. (C) *Miwi* expression was assessed by qPCR. Data were normalized to *actin* level and expressed in arbitrary units (a.u.) (mean ± SEM of three independent mice/group). **P < 0.05 (Student's *t*‐test).

To explore how Coprs regulates spermatocyte maturation, we next characterized Coprs protein interactors in testis by proteomics. After immunoprecipitation of Coprs in cell lysates prepared from testes of WT and *coprs* KO mice, and immunoprecipitate separation by SDS/PAGE and Colloidal Blue staining, we excised bands that were present in WT but not in KO extracts for mass spectrometry analysis (Fig. 2). Although Prmt5 and histone H4 are well‐characterized direct partners of Coprs 21, we did not identify out of the background any specific band in the range corresponding to Prmt5 molecular weight. Conversely and as expected, histones were differentially enriched in Coprs immunoprecipitates, validating the efficiency of the immunopurification procedure. Besides histones, Miwi was the most differentially enriched protein in Coprs immunoprecipitates, suggesting that Coprs is part of a Piwi complex (Fig. 2). Of note, this analysis identified also Stk31 (Tdrd8), a Tudor domain containing protein that interacts with Miwi and Mili upon Prmt5‐dependent methylation, but not critical for male germ cell development. IHC analyses of testis tissue sections showed that Miwi staining was lower in samples from *coprs* KO mice than WT littermates (Fig. 3A). Similarly, Miwi protein (western blotting) and mRNA (RT‐qPCR) levels were significantly reduced in testes from *coprs* KO mice compared with WT littermates (Fig. 3B,C).

**Figure 4:**
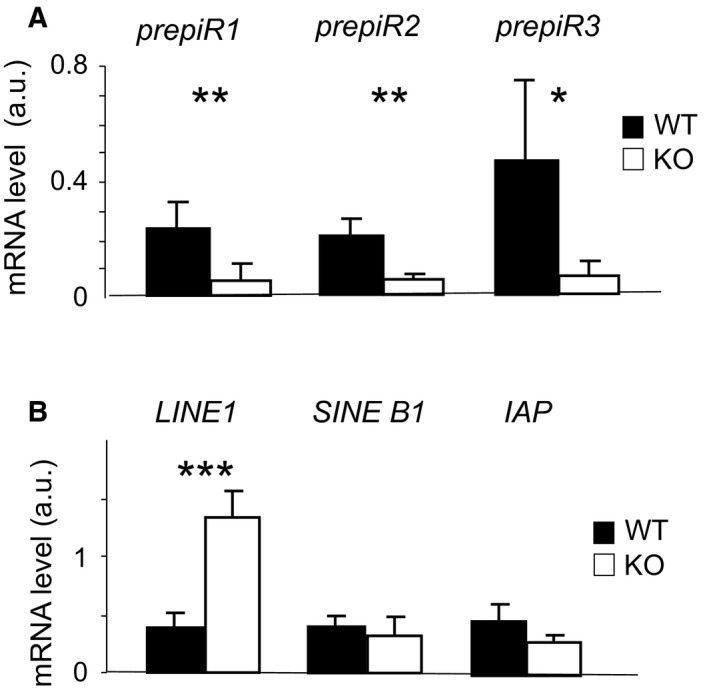
*coprs* KO deregulates the expression of pre‐piRNAs and *LINE1*. (A) The expression profile of the indicated pachytene piRNAs precursors (*prepiR1*,* prepiR2*, and *prepiR3*) was assessed by qPCR and normalized to *actin* level. Values are expressed in arbitrary units (a.u.) and are the mean ± SEM of three independent mice/group. (B) The RNA profile of the indicated transposons was assessed by qPCR and analyzed as in A); *P < 0.1; **P < 0.05; ***P < 0.001 (Student's *t*‐test).

As Miwi and piRNAs associate in a functional complex that controls genome integrity, we evaluated whether piRNAs expression was altered upon *coprs* gene ablation. RT‐qPCR analysis showed that pachytene piRNA precursors (pre‐piRNAs) 1, 2, and 3 were less abundant in *coprs* KO than WT testes (Fig. 4A). Although 75% of piRNAs expressed at the pachytene stage are not yet annotated, approximately 17% of them play a key role in inhibiting retrotransposon expression 32. Therefore, we used the RNA expression levels of the most abundant retrotransposons, namely *LINE1*,* SINE B1*, and *IAP*, as readout of potential dysfunction in the piRNA pathway. The transcript level of *LINE1,* but not of *SINE B1* and *IAP,* was upregulated in *coprs* KO compared to WT testis samples (Fig. 4B).

Altogether, these results suggest that Coprs is involved in the piRNA pathway and that its deficiency in male germ cells perturbs LINE1‐related genome surveillance mechanisms.

### Coprs and teratozoospermia

**Figure 5:**
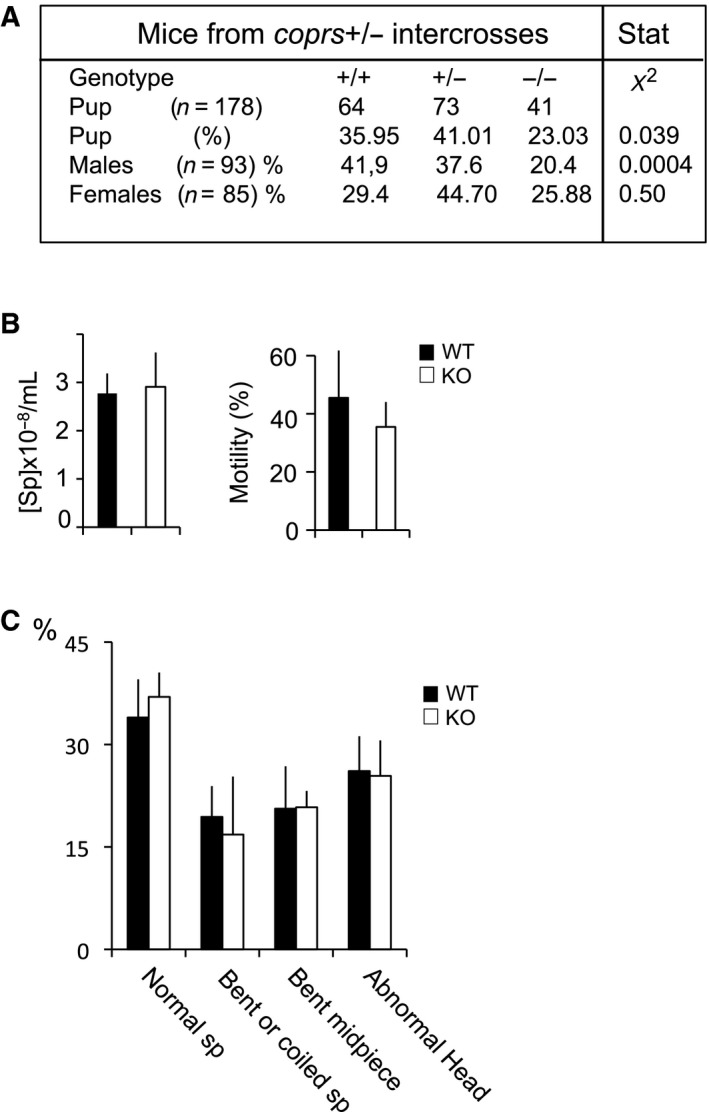
Non‐Mendelian inheritance of the *coprs*‐floxed allele and analysis of different parameters in spermatozoa of WT and *coprs* KO mice. (A) Table showing the relative proportion of animals obtained from intercrosses between heterozygous *coprs* mice. (B) Histograms showing the mean ± SD of spermatozoa concentration and motility in semen samples from WT (*n* = 3) and *coprs* KO (*n* = 3) mice at 11 weeks of age. (C) Quantification of the morphology of spermatozoa (sp) from the same semen samples analyzed in (B) and expressed in percent represents the mean ± SD.

**Figure 6:**
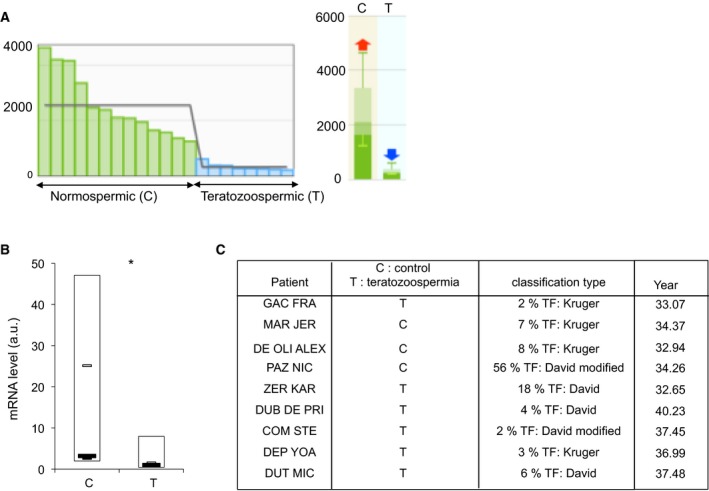
Low level of *Coprs* mRNA and teratozoospermia. (A) Transcription profiling of human sperm from individuals with normospermia (C: normally fertile) and teratozoospermia (T) (GEO data: E‐GEOD‐6872). (B) *Coprs* expression in semen mRNA samples from controls (C, *n* = 3) and patients with fertility problems (T, *n* = 6) was analyzed by RT‐qPCR. Data normalized to *HPRT* RNA and expressed in arbitrary units (a.u.) represent the mean ± SD; *P < 0.1 (Student's *t*‐test). (C) Clinical classification of each patient according to the % of typical spermatozoon forms (TF) evaluated using one of three classical methods, as indicated 33, 34, 35.

To evaluate whether the molecular alterations identified in *coprs* KO testes influenced reproduction, we first measured the mating efficiency of *coprs* KO males and control littermates. Several independent crosses of *coprs* heterozygous animals identified a non‐Mendelian inheritance of *coprs*‐floxed allele in males (Fig. 5A). Matings of *coprs* KO males with WT females were successful without noticeable reduction of litter size (data not shown), indicating that spermatocyte maturation impairment in these mice was not severe enough to impair fertility. Considering that several knockout mice with reduced sperm count or altered spermatogenesis, but normal fertility, have been described previously 33, we evaluated the number, motility, and morphology of spermatozoa in 11‐week‐old mice. We did not find any significant difference between WT and *coprs* KO mice (Fig. 5B,C). However, probing GEO data for biological situation of differential *Coprs* expression, low level of *Coprs* mRNAs correlated strongly with teratozoospermia, a human pathology linked to male infertility (Fig. 6A). Therefore, we conducted a pilot experiment to evaluate *Coprs* mRNA level by RT‐PCR in patients with teratozoospermia associated with fertility defects and confirmed that it was downregulated compared with patients without teratozoospermia (Fig. 6B,C).

## Discussion

Here, we show in mouse that Coprs is expressed in spermatogonia and that in its absence, maturation of spermatogonia during meiosis and spermiogenesis is slightly impaired. This leads to a significant accumulation of spermatocytes at the pachytene stage and a decrease of round/elongated spermatids. Many changes occur within the cell nucleus during spermatocyte maturation, including the programmed replacement of histones by a set of basic nuclear proteins such as histone variants and protamines to increase chromatin compaction, an essential event for male fertility. Consistent with an accumulation of cells in pachytene, the mRNA level of *H1t2,* a histone variant that is specifically expressed in pachytene spermatocytes*,* was increased in *coprs* KO cells. Moreover, the *Prm1*/*Prm2* ratio was increased in KO spermatocytes, a parameter that was reported as being associated with male infertility in humans 34, 35, 36. Our proteomic analysis identified Miwi as a potential Coprs interactor, although at this stage our data do not address whether this interaction is direct or through the presence of these two proteins within a common complex. Moreover, both Miwi protein and mRNA levels were significantly decreased in *coprs* KO mice, as well as the expression level of pachytene piRNAs. Altogether, these results suggest that the Miwi‐piRNA pathway is altered in *coprs* KO testis. How Coprs controls Miwi and piRNA levels remains unknown, and future studies will address this point. It can be hypothesized that the stability of Miwi protein or Miwi complexes requires Coprs. In addition, the reduced Miwi level in *coprs* KO testes, resulting *de facto* in a lower Miwi slicer activity, might impact on piRNAs that target active retrotransposons, such as LINE1, thus weakening the maintenance of transposon silencing 12.

In contrast to *Miwi* KO mice in which spermatogenesis is arrested at the round spermatid stage with no retrotransposon upregulation 14, *coprs* KO testes present only a slower spermatogenesis progression but showed a significant derepression of *LINE1 expression*. This suggests that the low but detectable level of Miwi present in *coprs* KO animals is sufficient to ensure spermatogenesis and that Coprs could contribute to the control of *LINE1* expression and genome stability beyond its impact on Miwi expression. A similar mild phenotype, that is, normal fertility and viability associated with a derepression of *LINE1* transcripts), was described in mice deficient for *Exd1* which encodes for a partner of Miwi2 piRNA biogenesis factor TDRD12 37. Importantly, the functional consequences of Exd1 loss in testis, that is, massive derepression of *LINE1* elements and an arrest in spermatogenesis, are revealed only in the *Exd1* ^*−/−*^ *;Tdrd12* ^*+/−*^ genetic background 38. As well, we can make the assumption that a stronger impact of Coprs depletion on spermatogenesis might be observed by breeding *coprs* mice with animals in which other Piwi‐associated protein‐encoding genes have been invalidated. However, we cannot exclude on the basis of the non‐Mendelian inheritance of the *coprs‐*floxed allele that the more severe *coprs* KO phenotypes might affect early embryo viability. This could explain how *coprs* absence led to an apparent mild phenotype compared with that of Miwi and Miwi‐associated proteins. Thus, it is possible that KO mice upon F2 and/or the following generations might affect more strongly the reproduction parameters and give rise to a phenotype reminiscent of human teratozoospermia, a pathology associated with low *Coprs* expression level. Although this conclusion requires confirmation in a larger cohort of patients, these data support the hypothesis that *Coprs* is a candidate diagnostic marker for teratozoospermia.

## Conflict of interest

The authors declare no conflict of interest.

## Authors contributions

PC executed and analyzed FACS experiments; HD carried out immunohistochemistry experiments; DH and SH provided human samples and critical discussions of the manuscript; SC provided critical discussions of the manuscript; and FE carried out PCR data analysis, designed the study, and drafted the manuscript.

## Supporting information


**Fig. S1.** (A) Western blot detection of whole cell extracts from WT (*n* = 4) and *coprs* KO (*n* = 3) testes with antibodies against the indicated proteins. The expression of Prmt5, a Coprs partner, was comparable in KO and WT samples. (B) *Coprs* RNA expression level from RNA‐seq data in different human tissues (GEO data) is expressed as TPM (transcripts per million), i.e., the mean values of the different samples from each tissue. Sample color‐coding is based on tissue groups with common functional features.
