# Erratum to: Coprs inactivation leads to a derepression of LINE1 transposons in spermatocytes

**DOI:** 10.1002/2211-5463.12607

**Published:** 2019-02-28

In the paper by Paul *et al*. 1, the authors’ names were reversed.

The correct author listing is:

Conception Paul^1^, Hélène Delpech^2^, Delphine Haouzi^3^, Samir Hamamah^3^, Claude Sardet^2^ and Eric Fabbrizio^2^

The error has been corrected on the original article. The publishers apologise for any confusion.
