# Supplementary material for: Coprs inactivation leads to a derepression of LINE1 transposons in spermatocytes
Source: FEBS Open Bio. 2018 Dec 19;9(1):159–68. doi: 10.1002/2211-5463.12562 (PMC6325579; doi:10.1002/2211-5463.12562)
Supplement: Supplementary file 1 — Fig. S1. (A) Western blot detection of whole cell extracts from WT (n = 4) and coprs KO (n = 3) testes with antibodies against the indicated proteins. The expression of Prmt5, a Coprs partner, was comparable in KO and WT samples. (B) Coprs RNA expression level from RNA‐seq data in different human tissues (GEO data) is expressed as TPM (transcripts per million), i.e., the mean values of the different samples from each tissue. Sample color‐coding is based on tissue groups with common functional features. [file FEB4-9-159-s001.docx]

Figure S1: A) Western blot detection of whole cell extracts from WT (n=4) and *coprs* KO (n=3) testes with antibodies against the indicated proteins. The expression of Prmt5, a Coprs partner, was comparable in KO and WT samples. B) *Coprs* RNA expression level from RNA-seq data in different human tissues (GEO data) is expressed as TPM (transcripts per million), i.e., the mean values of the different samples from each tissue. Sample color-coding is based on tissue groups with common functional features.


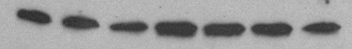


Tbp


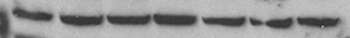


Prmt5


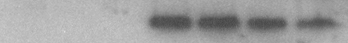


Coprs

A

WT

KO

1 2 3 4 1 2 3


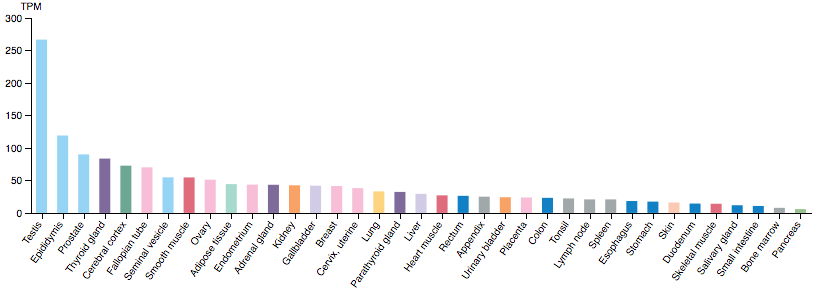


B
